# Application of the thermofluor PaSTRy technique for improving foot-and-mouth disease virus vaccine formulation

**DOI:** 10.1099/jgv.0.000462

**Published:** 2016-07

**Authors:** Abhay Kotecha, Fuquan Zhang, Nicholas Juleff, Terry Jackson, Eva Perez, Dave Stuart, Elizabeth Fry, Bryan Charleston, Julian Seago

**Affiliations:** ^1^​ Division of Structural Biology, Wellcome Trust Centre for Human Genetics, University of Oxford, Roosevelt Drive, Oxford OX3 7BN, UK; ^2^​ The Pirbright Institute, Woking, Surrey, GU24 0NF, UK

**Keywords:** FMDV, capsid stability, PaSTRy, vaccine production

## Abstract

Foot-and-mouth disease (FMD) has a major economic impact throughout the world and is a considerable threat to food security. Current FMD virus (FMDV) vaccines are made from chemically inactivated virus and need to contain intact viral capsids to maximize efficacy. FMDV exists as seven serotypes, each made up by a number of constantly evolving subtypes. A lack of immunological cross-reactivity between serotypes and between some strains within a serotype greatly complicates efforts to control FMD by vaccination. Thus, vaccines for one serotype do not afford protection against the others, and multiple-serotype-specific vaccines are required for effective control. The FMDV serotypes exhibit variation in their thermostability, and the capsids of inactivated preparations of the O, C and SAT serotypes are particularly susceptible to dissociation at elevated temperature. Methods to quantify capsid stability are currently limited, lack sensitivity and cannot accurately reflect differences in thermostability. Thus, new, more sensitive approaches to quantify capsid stability would be of great value for the production of more stable vaccines and to assess the effect of production conditions on vaccine preparations. Here we have investigated the application of a novel methodology (termed PaSTRy) that utilizes an RNA-binding fluorescent dye and a quantitative (q)PCR machine to monitor viral genome release and hence dissociation of the FMDV capsid during a slow incremental increase in temperature. PaSTRy was used to characterize capsid stability of all FMDV serotypes. Furthermore, we have used this approach to identify stabilizing factors for the most labile FMDV serotypes.

## Introduction

Foot-and-mouth disease (FMD) affects cloven-hoofed animals, has a major economic impact globally and is a considerable threat to food security. Vaccination remains the most effective approach for controlling FMD. However, FMD virus (FMDV) exists as seven serotypes (A, O, C, Asia 1 and South African Territories SAT 1, SAT 2 and SAT 3), and each is formed by numerous constantly evolving subtypes. This greatly complicates control as vaccines to one serotype do not protect against the others and antigenic variation within each serotype can severely limit cross-immunity. Thus the most effective vaccines need to closely match to the outbreak virus and as a result there is a constant need to develop new vaccine strains.

FMDV is non-enveloped and the capsid is formed from 60 copies each of four structural proteins [VP4 (1A), VP2 (1B), VP3 (1C) and VP1 (1D)] that encase a single-stranded, positive-sense RNA genome. The capsid proteins form a pseudo T=3 icosahedral structure. VP1 is located at the fivefold axes of symmetry; VP2 and VP3 alternate around the threefold axes while VP4 is internal. Capsid assembly proceeds through a number of intermediate stages: a single copy each of VP0 (the precursor of VP4 and VP2), VP1 and VP3 form the monomeric subunit (protomer). Five protomers then self-assemble to form penta-meric subunits (12S subunits), and 12 pentamers associate with the viral RNA while undergoing a maturation cleavage to convert VP0 into VP2 and VP4 to form the mature virions (146S particles).

Current FMDV vaccines are made from chemically inactivated virus and need to contain intact viral capsids for maximum efficacy. FMDV capsids are both pH and temperature labile and readily dissociate into 12S subunits under mild acidic conditions and even at mildly elevated temperatures (Doel & Baccarini, 1981; Kotecha* et al.*, 2015). Furthermore, the different serotypes exhibit some variation in capsid thermostability with the O and SAT serotypes being particularly sensitive to temperature. Chemical inactivation of FMDV increases capsid instability (Doel & Baccarini, 1981), which further reduces vaccine efficacy. The thermal lability of FMD vaccines necessitates the requirement of a cold chain from manufacture to use in the field, and this is often unfeasible in endemic regions during vaccine administration. At present, there are a limited number of approaches that can be used to analyse capsid thermostability, such as ELISA-based methodologies and comparative infectivity assays, but these are relatively time consuming, cannot be applied to all serotypes and cannot identify subtle differences in capsid stability. Thus, new methodologies are needed to more accurately determine FMDV capsid thermostability. These methods could help with the selection of less-labile vaccine seed stocks, optimization of vaccine production and rapid monitoring of vaccine quality in the field. Thermofluor assays (also known as thermal shift assays or differential scanning fluorimetry, DSF) have been used to characterize the thermostability of a diverse range of proteins for the identification of optimal crystallization formulations (Ericsson* et al.*, 2006; Vedadi* et al.*, 2006), the detection of low-molecular-mass ligands that confer increased stability (Niesen* et al.*, 2007), and for assessing protein quality and aggregation (Mezzasalma* et al.*, 2007; Senisterra & Finerty, 2009). Recently, thermofluor methodology was adapted to develop a novel, plate-based thermal release assay (termed PaSTRy) to analyse capsid stability of non-enveloped picornaviruses (e.g. poliovirus, equine rhinitis A virus and bovine enterovirus type 2) (Walter* et al.*, 2012). Here, we have used PaSTRy to characterize the capsid stabilities of viruses representative of all of the FMDV serotypes and used the most thermal-labile serotypes, namely O, SAT1, SAT2 and SAT3, to determine conditions that influence capsid stability. In addition, we have investigated the potential of PaSTRy to monitor capsid stability during different stages of vaccine production and storage.

## Results

### PaSTRy analysis of FMDV capsids

PaSTRy can use two different dye-based methodologies to monitor capsid dissociation during a slow incremental increase in temperature. Capsid disassembly can be detected using either (i) a dye that is sensitive to the hydrophobic regions normally concealed in the native capsid but exposed during dissociation (the hydrophobicity assay) or, alternatively, (ii) a dye that is sensitive to the viral genome, which is released during capsid dissociation (RNA release assay). In our assays, SYPRO orange dye was used to monitor protein unfolding and SYBR green II dye to monitor RNA release. Assays were carried out using a quantitative (q)PCR machine and samples were exposed to a recurrent increase in temperature from 25 °C to 94 °C.

**Fig. 1.**
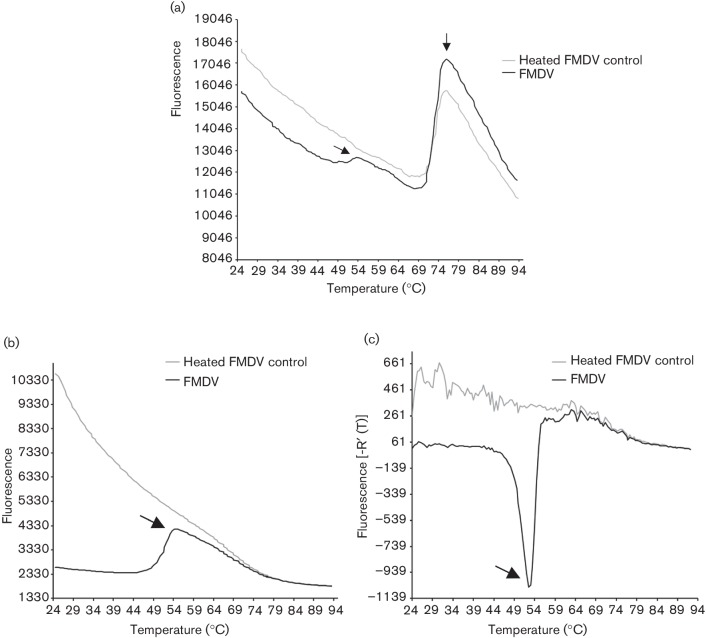
FMDV capsid integrity can be monitored by PaSTRy. (a) Dissociation curves performed at pH 7.4 for pre-heated (56 °C for 10 min) or non-heated FMDV (O1M), determined by hydrophobicity assay which detects unfolding of capsid proteins over a temperature gradient. Arrows indicate two separate dissociation peaks in the non-heated sample with corresponding* T*_m_ of 52.5 °C and 73.5 °C. (b) Dissociation curves performed at pH 7.4 for pre-heated (56 °C for 10 min) or non-heated FMDV (O1M), determined by RNA release assay, which detects release of viral genome over a temperature gradient. Arrow indicates a single dissociation peak with a corresponding *T*_m _of 52 °C. (c) Data in (b) presented as a negative first-derivative plot.

Initially, we focused on determining which dye was most applicable to monitor FMDV capsid dissociation using PaSTRy. To perform the assays, purified virus was prepared by sucrose gradient ultracentrifugation. Sucrose has been shown to stabilize picornaviruses, including FMDV, so the assays were performed in sufficient volumes to dilute the concentration of sucrose to <2 % (w/v) (Rombaut* et al.*, 1994; Terry* et al.*, 1983; Walter* et al.*, 2012). The first assays utilized infectious FMDV [O1 Manisa (O1M) serotype] and monitored protein unfolding (i.e. the hydrophobicity assay) in PBS at pH 7.4, a physiological pH environment in which FMDV capsids are relatively stable. As a positive control for capsid dissociation, virus was heated at 56 °C for 10 min prior to performing the assay. Fig. 1(a) shows a typical dissociation curve achieved using 0.4 µg of virus. The virus sample that was not pre-heated exhibited two separate melting temperatures (*T*_m_): a major* T*_m_ at 73.5 °C and a less prominent *T*_m_ at 52.5 °C, whilst the pre-heated virus exhibited only a single *T*_m_ at 73 °C.

Next, we used PaSTRy to detect viral RNA to determine the temperature (*T*_r_) at which the viral genome is released from infectious particles. Fig. 1(b) shows a typical dissociation curve achieved using 0.4 µg of virus to perform the assay, and Fig. 1(c) shows the same data but as a negative first-derivative plot (the format used to present PaSTRy data from here onwards). In contrast to the hydrophobicity assays, a single clear *T*_r_ (52 °C) was observed, corresponding to the less prominent *T*_m_ of 52.5 °C produced in the hydrophobicity assay. Based on these results, the RNA release assay was selected to monitor viral stability in all further experiments.

### Analysis of pH on capsid stability

Next we analysed the effect of pH on thermal stability of FMDV capsids using the RNA release assay. Walter* et al.* (2012) previously showed that the SYBR green II dye can be used for PaSTRy across a broad pH range without significant changes to the spectral properties of the dye or sufficient loss of assay efficiency. FMDV dissociates rapidly under mild acidic conditions, a process which is enhanced by increases in temperature. For our analysis we used viruses representing FMDV serotypes that are particularly thermolabile, namely O, SAT1, SAT2 and SAT3. The assay was carried out using purified viruses at pH 7, 7.4, 8, 8.5 and 9. Table 1 lists the *T*_r _values determined and shows that the capsids of all serotypes exhibited optimal thermostability at pH 8.

**Table 1.** Effect of pH on the thermostability of O (O1M), SAT1 (KNP 196/91/1), SAT2 (Zim 7/83) and SAT3 (KNP 1/08/3) FMDV using RNA release assays The respective temperature at which the RNA genome was released [*T*_r_ (°C)] at each pH value is shown.

FMDV serotype	pH 7	pH 7.4	pH 8	pH 8.5	pH 9
O	45.2±0.2	52±0.0	54±0.0	51.5±0.0	49±0.1
SAT1	43.6±0.3	44±0.1	45±0.2	44.5±0.1	43.8±0.2
SAT2	50.5±0.1	51±0.1	52.5±0.2	51±0.0	50±0.1
SAT3	49±0.2	48.5±0.0	51.5±0.1	48.5±0.3	48.5±0.1

### The effect of cations, ammonium ions and sucrose on FMDV stability

**Fig. 2.**
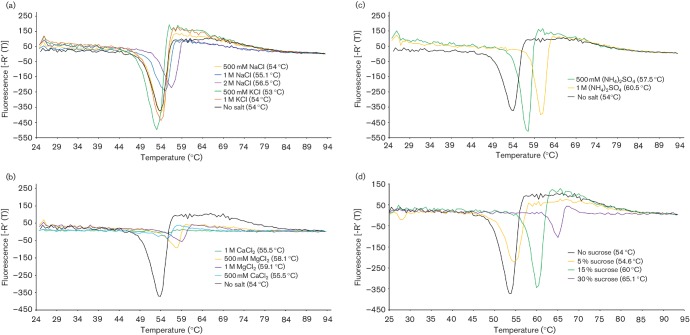
PaSTRy facilitates identification of FMDV capsid stabilizing factors. Dissociation curves for FMDV (O1M) determined by RNA release assay performed at pH 8 in the presence of (a) monovalent cations (NaCl and KCl), (b) divalent cations (CaCl_2_and MgCl_2_), (c) (NH_4_)_2_SO_4 _and (d) sucrose at the concentrations indicated. The respective temperatures of genome release, and hence capsid disassembly, are shown in parentheses. Results are representative of three independent experiments.

Next, a series of assays was carried out to investigate the effect of different cations on the stability of infectious FMDV. As the optimal pH for capsid stability was pH 8.0 (see above), these assays were carried out at this pH. As a preliminary screen, sodium and potassium (monovalent cations), and calcium and magnesium (divalent cations) were tested using purified O1M. Fig. 2(a, b) shows that sodium (>1 M), calcium (>0.5 M) and magnesium (>0.5 M) cations enhanced FMDV thermostability in comparison with the control (1 M CaCl_2_, *T*_r_ 55.5 °C; 2 M NaCl, *T*_r_ 56.5 °C; 1 M MgCl_2_, *T*_r_ 59.1 °C; control, *T*_r_ 54 °C). Similar assays were also performed to investigate the effects of ammonium ions and sucrose on capsid stability. Fig. 2(c, d) shows that ammonium ions also enhanced FMDV thermostability [1 M (NH_4_)_2_SO_4_, *T*_r_ 60.5 °C], and the addition of sucrose conferred the greatest stability of the reagents tested (30 % sucrose, *T*_r_ 65.1 °C).

**Fig. 3.**
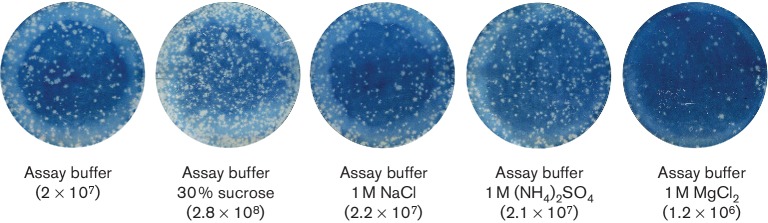
The presence of sucrose in FMDV samples leads to enhanced levels of infection. FMDV (O1M) was incubated for 16 h at room temperature in the presence of the stabilizing factors determined by PaSTRy [NaCl and MgCl_2_, (NH_4_)_2_SO_4 _and sucrose] at the concentrations indicated. Plaque assays were then performed to determine viral titres (p.f.u. ml^-1^, shown in parentheses). Results are representative of three independent experiments.

To confirm the stabilizing effect of the above reagents, infectivity assays were carried out following prolonged (16‌ h) exposure of the virus (O serotype) to 1‌ M Na^+^, 1‌ M Mg^2+^, 1‌ M NH_4_^+^ or 30 % sucrose. The control virus was incubated for the same period of time but not exposed to the respective factors. In comparison with the control (titre, 2×10^7 ^p.f.u. ml^-1^), a reduced titre was observed for virus incubated in the presence of magnesium ions (1 M MgCl_2_, 1.2×10^6^ p.f.u. ml^−1^) (Fig. 3). In contrast, exposure to sodium ions (1 M NaCl, 2.210^7^ p.f.u. ml^−1^), ammonium ions [1 M (NH_4_)_2_SO_4_, 2.1×10^7^ p.f.u. ml^−1^] or sucrose (30 % sucrose, 2.8×10^8^ p.f.u. ml^−1^) led to an increase in virus titres. Statistical analyses (one-way ANOVA followed by a Dunnett multiple comparisons test with control) indicated there was a significant increase in titre when sucrose was used (*P-*value 0.000; 95 % confidence interval, CI).

Having identified a panel of stabilizing factors (1 M Na^+^, 1 M Mg^2+^, 1 M NH_4_^+^, 30 % sucrose) for type O FMDV, their effects on the stability of SAT1, SAT2 and SAT3 viruses were also investigated by using RNA release assays. Table 2 lists the respective* T*_r_ values determined for each virus. In agreement with the data generated using O1M, most of the stabilizing factors also led to increases in the stability of the SAT1, SAT2 and SAT3 viruses in comparison with the respective control assays. The one exception was a reduction in thermostability of SAT2 in the presence of magnesium cations (1 M MgCl_2_, *T*_r_ 48 °C). Interestingly, the presence of sodium ions resulted in the greatest increase in thermostability for both SAT1 (1 M NaCl, *T*_r_ 50.6 °C) and SAT2 (1 M NaCl, *T*_r_ 58 °C), whilst sucrose resulted in the greatest increase in thermostability for SAT3 (30 % sucrose, *T*_r_ 55.5 °C).

**Table 2.** Effect of cations and sucrose on the thermostability of SAT1 (KNP 196/91/1), SAT2 (Zim 7/83) and SAT3 (KNP 1/08/3) FMDV using RNA release assays The respective temperature at which the RNA genome was released [*T*_r_ (°C)] is shown.

Virus	200 mM NaCl, pH 8	1 M NaCl, pH 8	1 M (NH_4_)_2_SO_4_, pH 8	1 M MgCl_2_, pH 8	30 % Sucrose, pH 8
SAT1	45±0.1	50.6±0.2	47±0.2	49±0.3	49±0.1
SAT2	52.5±0.0	58±0.1	55±0.3	48±0.2	57.5±0.2
SAT3	51.5±0.1	53±0.0	54.6±0.3	54.5±0.2	55.5±0.0

### Analysis of inactivated FMDV

**Fig. 4.**
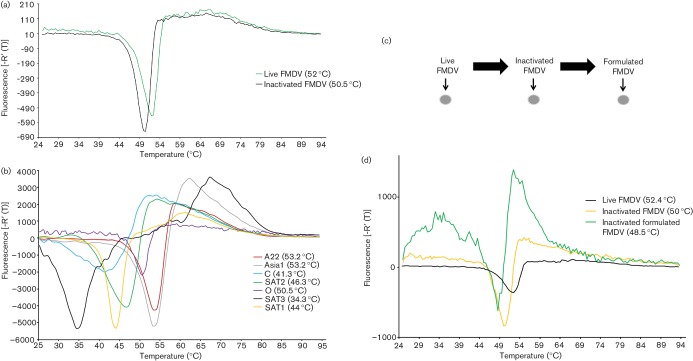
PaSTRy facilitates comparative thermostability analysis of FMDV capsids from different serotypes and during vaccine production. (a) Dissociation curves determined by RNA release assay for purified FMDV before and after inactivation. (b) Dissociation curves determined by RNA release assay for purified representatives of the FMDV serotypes (SAT1 KNP 196/91/1, SAT2 Zim/7/83, SAT3 KNP, O1M, A22 Iraq, C Oberbayern and Asia1 Shamir) following inactivation. (c) Schematic of the three different stages of small-scale FMD vaccine (O1M) production. Unpurified virus samples from each stage were used to assess capsid stability by PaSTRy as shown in (d). (d) Dissociation curves determined by RNA release assays for unpurified live FMDV, unpurified inactivated FMDV and unpurified formulated FMDV. The respective temperatures of genome release, and hence capsid disassembly, are shown in parentheses. Results are representative of three independent experiments.

The above analyses were carried out using infectious virus; however, the current FMD vaccines are made by inactivating virus chemically, a procedure which has previously been shown to reduce capsid thermostability (Doel & Baccarini, 1981). Therefore, we also investigated release of the viral RNA genome using purified virus samples that had undergone two rounds of chemical inactivation (inactivated virus). Fig. 4(a) shows that RNA release assays carried out at pH 7.4 with inactivated virus (O1M) resulted in a single dissociation event, although, as expected, a lower *T*_r_ (50.5 °C) was seen compared with the parental infectious virus (52 °C). To show the technique can be used on other inactivated FMDV isolates, assays using representative strains of all seven serotypes were performed in parallel. Fig. 4(b) shows the relative stabilities for each serotype, and the *T*_r_ were calculated as SAT1, *T*_r_ 44 °C; SAT2, *T*_r_ 46.3 °C; SAT3, *T*_r_ 34.3 °C; O, *T*_r_ 50.5 °C; A22, *T*_r_ 53.2 °C; C, *T*_r _41.3 °C; Asia1, *T*_r_ 53.2 °C.

Having shown that specific cations can increase the thermostability of infectious FMDV, we postulated cations would also enhance the thermostability of inactivated viruses. In addition, we aimed to improve upon our earlier experiments and test combinations of cation concentrations for their capacity to enhance thermostability. To facilitate this, RNA release assays (using purified and inactivated O1M) were performed using a series of buffered solutions containing different concentrations of sodium (0, 150, 200 and 400  mM) in combination with different concentrations of magnesium, manganese or calcium (2, 10 and 50 mM). Table 3 lists the *T*_r_ values derived from the screen. At low concentrations (2 mM) of magnesium or manganese, adding sodium to 150 mM had a stabilizing effect; however, further increasing the concentration of sodium did not enhance stability. In contrast, no increase in stability was observed at low concentrations of calcium (2 mM) when sodium (150 mM–400 mM) was added. Interestingly, two *T*_r_ values were obtained from all assays performed in the presence of higher concentrations of manganese (10 mM and 50 mM) and calcium (50 mM), showing that these conditions did not confer an equal increase in stability to all particles within the same sample. With regards to assays that produced data showing a single dissociation curve, and hence an equal increase in stability, the highest *T*_r_ values (57.2 °C) were obtained using 10 mM calcium in combination with sodium (0.2–0.4 mM). Together, these analyses show that combinations of cations at specific concentrations can enhance capsid stability.

**Table 3.** Effect of cations on the thermostability of inactivated O1M FMDV using RNA release assays The respective temperature at which the RNA genome was released [*T*_r_ (°C)] at each cation concentration is shown. Optimal *T*_r_ values (>57 °C) obtained using cation conditions that produced a single dissociation curve are shown in bold type. nd, Not determined.

		NaCl concentration			
		0 mM	150 mM	200 mM	400 mM
		nd	50.6±0.2	nd	nd
MgCl_2_ conc.	2 mM	43.2±0.2	46.3±0.2	45.3±0.0	45.2±0.3
	10 mM	53.2±0.2	53.2±0.2	53.2±0.1	53.2±0.2
	50 mM	53.2±0.3	54.2±0.4	53.2±0.2	53.2±0.1
MnCl_2_ conc.	2 mM	49.2±0.3	50.2±0.4	49.2±0.2	49.2±0.0
	10 mM	49.3±0.3; 57.2±0.3	50.3±0.0; 57.3±0.1	50.3±0.2; 57.2±0.3	52.2±0.1; 56.2±0.0
	50 mM	46.2±0.4; 56.2±0.1	46.2±0.3; 56.2±0.0	46.2±0.5; 56.2±0.2	47.2±0.5; 55.2±0.3
CaCl_2_ conc.	2 mM	53.2±0.4	53.2±0.0	53.2±0.2	52.2±0.0
	10 mM	55.2±0.2	55.2±0.3; 58.2±0.6	**57.2**±0.5	**57.2**±0.3
	50 mM	53.2±0.5; 57.2±0.3	53.2±0.6; 57.2±0.3	53.2±0.3; 57.2±0.0	55.2±0.3; 57.2±0.3

### Analysis of capsid stability during vaccine production and storage

Purification of FMDV and vaccine formulation are both time consuming and costly and it would be of benefit if capsid integrity could be monitored during, rather than at the end of, vaccine preparation. PaSTRy offers a potential technique for analysing thermostability of FMDV during the vaccine manufacturing process. To determine this, a small-scale FMD vaccine preparation was prepared for FMDV (O1M). Samples taken from different stages of the preparation [live virus, inactivated virus and inactivated virus formulated with Montanide ISA 201(FMD vaccine)] were first concentrated and then anlaysed by PaSTRy (Fig. 4c, d). To confirm that the concentration protocol did not alter capsid stability by co-concentration of stabilizing factors, PaSTRy assays were performed on concentrated virus that had undergone buffer exchange and in comparison to non-buffer-exchanged virus no differences in* T*_r_ values were observed (data not shown). Fig. 4(d) shows that capsid dissociation during different stages of small-scale vaccine production could be monitored by PaSTRY using the RNA release assay. Interestingly, the capsids of inactivated FMDV adjuvanted with Montanide ISA 201 exhibited a reduced dissociation temperature in comparison to unformulated inactivated virus (*T*_r_ 48.5 °C and 50 °C, respectively) (Fig. 4d).

Next we determined whether PaSTRY can be used to investigate the effects of virus storage on the capsid of unformulated and formulated (with either Montanide ISA 201 or 206 adjuvant) inactivated virus. To do this, unformulated and formulated inactivated FMDV (O 77/78) samples were stored for up to 21 days at 4 °C, or for 10 days at 4 °C followed by 2 days at 37 °C. Table 4 summarizes the results obtained. The storage of unformulated inactivated virus (*T*_r_ 50.5 °C at Day 0) under either condition led to a gradual decrease in capsid stability. As expected, removal of the unformulated inactivated virus from cold chain conditions (4 °C) after 10 days and exposure to 37 °C for 2 days led to a lower *T*_r_ value (45.6 °C) in comparison with continued storage at 4 °C for a longer period (21 days) (*T*_r_ 48.5 °C). Compared with the unformulated vaccine, the presence of either adjuvant led to an initial reduction of dissociation temperature. Following exposure of the Montanide ISA 206-formulated vaccine to 37 °C for 2 days, a dissociation curve was unattainable, presumably due to capsid dissociation. Interestingly, capsid thermostability did not decline further in the presence of Montanide ISA 201 under either storage condition, suggesting this adjuvant can inhibit capsid dissociation during storage.

**Table 4.** Effect of storage temperature on thermostability of unformulated and formulated (Montanide ISA 201 or Montanide ISA 206) O 77/78 FMD vaccine The respective temperature at which the RNA genome was released [*T*_r_ (°C)] under each storage condition is shown. nd, Not determined.

	Day 0 (4 °C)	10 days (4 °C)	21 days (4 °C)	10 days (4 °C) 2 days (37 °C)
Unformulated	50.5±0.0	50.6±0.3	48.5±0.2	45.6±0.3
Montanide ISA 201	47.1±0.3	47.5±0.1	48±0.1	47.6±0.2
Montanide ISA 206	49±0.1	47.5±0.2	45.5±0.4	nd

## Discussion

To characterize the thermostability properties of the different FMDV serotypes we used PaSTRy analysis to monitor capsid dissociation under a range of conditions. Two versions of the qPCR-based assay were initially tested using fluorescent dyes that physically interact with virion components that are exposed following capsid dissociation; one detects the exposure of hydrophobic regions that are normally hidden in the capsid and the other the release of the RNA genome. When exposure of hydrophobic residues was monitored, three times more virus was required, compared with similar assays monitoring release of the RNA genome, to visualize a dissociation curve corresponding to the approximate temperature (~50 °C) at which FMDV (O serotype) is known to dissociate (Terry* et al.*, 1983). Furthermore, at least two *T*_m_ (52.5 °C and 73.5 °C) were observed for each virus in the hydrophobicity assay. The less prominent *T*_m_ was coincident with the release of RNA (*T*_r_ 52 °C), suggesting that it resulted from exposure of the inner surfaces of capsid as a result of pentamer dissociation. The second, more prominent *T*_m_ most likely results from heat denaturation of dissociated pentamers. These results are consistent with those obtained using PaSTRy analysis for the related picornaviruses, BEV2 and poliovirus (Walter* et al.*, 2012). Identification of two dissociation peaks in the hydrophobicity assays raises the possibility of using this methodology to study the conformational changes that occur to the FMDV capsid prior to release of the genome. Interestingly, two *T*_r_ values were also observed when RNA release assays were performed in the presence of specific divalent cations (manganese and calcium). To date, the role of manganese and calcium on capsid unpackaging within host cells has not been characterized.

The FMDV capsid is both pH and temperature labile and dissociates under mild acidic conditions and mildly elevated temperatures. Inactivation of FMDV during vaccine production renders FMDV more susceptible to heat-induced dissociation, which necessitates the need for stringent temperature control during manufacture and for a cold chain to the point of inoculation. This is important because the dissociation of viral particles in FMD vaccines affects their efficacy and the ability of vaccinates to generate adequate levels of neutralizing antibodies. There is a need for improved assays that can be used to monitor capsid integrity of vaccine candidate seed strains under identical conditions. This could facilitate the selection of the most stable circulating field strains, as well as the comparison of such strains with their recombinant counterparts. The RNA release assay facilitates these comparisons, and application of the assay at additional stages of vaccine manufacture offers the potential to improve the process and ultimately contributes towards improved FMD vaccines. Herein, we have shown that the RNA release assay can be used to accurately determine the thermostability of non-purified and purified FMDV in a range of environments and to investigate the stabilizing effects imported by specific factors on the capsid. Furthermore, the RNA release assay is applicable to all serotypes of FMDV, whether live or inactivated, and in the presence of routinely used water-in-oil in water adjuvants such as Montanide ISA 201 and Montanide ISA 206.

Our vaccine storage experiments showed that formulation with Montanide ISA 201 led to a reduction in capsid dissociation in comparison to formulation with Montanide ISA 206 and the no adjuvant control. Interestingly, Dar* et al.* (2013) have shown that Montanide ISA 201-adjuvanted FMD vaccine induces enhanced immune responses and protective efficacy in cattle in comparison with ISA 206 or GAHOL- adjuvanted vaccine. Montanide ISA 201 comprises a specific enriched light mineral oil and an extremely refined emulsifier obtained from mannitol and purified oleic acid of vegetable origin; however, it remains to be established whether any of these have stabilizing properties.

In this study we gave particular focus to investigating the respective thermostabilities of the least stable FMDV serotypes, namely O and the SAT viruses (SAT1–3). For the representatives of these serotypes tested, optimal capsid stability was observed at pH 8 and in the presence of either 30 % sucrose (O and SAT3 serotypes) or 1 M sodium chloride (SAT1 and SAT2). Sucrose has previously been shown to enhance the thermostability of FMDV, other related picornaviruses and also disparate viruses and has been shown to increase the stability of numerous vaccines. For example, Doel and colleagues showed that the thermostability of FMDV (O-BFS 1860) was approximately 20 % greater in the presence of 10 % sucrose in comparison with its stability in 2.5 % sucrose (Doel & Baccarini., 1981). Of note, the same authors that stated that optimal capsid stability was observed at pH 8. Their experiments involved the use of a cumbersome protocol to first heat the samples and subsequently analyse particle content using sucrose density gradient ultracentrifugation, and as such could not be used to quantify the amount of capsid dissociation that occurred between treatment and analysis. In contrast, the RNA release assay facilitates real-time analysis and can be performed using non-purified samples (data not shown). Other examples include the use of sucrose as a protectant of respiratory syncytial virus following nebulization and freeze-thawing, preventing unacceptable losses in viral infectivity (Grosz* et al.*, 2014). With regards to vaccines, sucrose has been shown to stabilize Sabin live polio vaccine (Mirchamsy* et al.*, 1978; Srivastava, 1989), live *peste des petits ruminants* vaccine (Riyesh* et al.*, 2011) and live flavivirus vaccines (Adebayo* et al.*, 1998; Wiggan* et al.*, 2011).

Thermal lability of FMDV at non-acidic pH is a consequence of the tenuous electrostatic and hydrophobic interactions between interpentameric subunits of the capsid. Presumably the enhanced stability seen in the presence of sodium and ammonium ions was due to screening of electrostatic repulsions on the surface of the capsid. In our hands, although higher concentrations of divalent cations (0.5 M and 1 M CaCl_2_ and MgCl_2_) appeared to increase the thermostability of the virus, their presence led to a reduction in the size of the dissociation curve (see Fig. 2b); this was not observed at lower concentrations (<50 mM), which also conferred stability (Table 3 and data not shown) and in part may be due to quenching of the fluorescent dye. However, plaque assays showed a reduction in titre following incubation of virus in the presence of high magnesium concentrations (1 M MgCl_2_) for 16 h, suggesting that prolonged exposure to the cation either destabilizes the virus or inhibits cell entry per se.

In summary, we have successfully shown that PaSTRy analysis can be used to accurately quantify the thermostability properties of all FMDV serotypes under a range of conditions, and as such, the application of this technique could benefit both vaccine production and research.

## Methods

### Cell culture and virus stocks.

A goat epithelium cell line, expressing the principle FMDV receptor (integrin αvß6), was subsequently used to passage the tagged viruses (P1) (Brehm* et al.*, 2009). Cells were infected for 24 h between passages.

### Virus purification.

Following cytopathic effect (CPE) of infected goat epithelium cells, virus within clarified supernatants was precipitated with a saturated solution of ammonium sulphate. Precipitated pellets were resuspended in PBS and pelleted over 30 % sucrose cushions by centrifugation at 104 000 *g* for 2.5 h at 12 °C. Pellets were resuspended in PBS/0.5 % (v/v) IGEPAL CA-630 (Sigma Aldrich), overlayed onto a 15–30 % sucrose gradient and then fractionated by centrifugation at 104 000 *g* for 3 h at 12 °C. The concentration of virus in each fraction was determined by measuring the absorbance at 260 nm.

### Virus concentration.

Non-purified virus samples were concentrated approximately 100-fold by the addition of 7.5 % PEG 6000 (w/v) before analysis by PaSTRy.

### Plaque assay.

Confluent monolayers of goat epithelium or BHK-21 cells were infected with serial dilutions of virus stocks, overlaid with indubiose and incubated for 24–48 h at 37 °C. Cells were fixed and stained (4 % formaldehyde in PBS containing methylene blue), and the overlay removed (Jackson* et al.*, 2000).

### Virus inactivation.

Chemical inactivation followed standard operating procedures in line with disease security regulations at The Pirbright Institute. Upon CPE, clarified supernatant was inactivated by two consecutive incubations with BEI (binary ethyleneimine at a final concentration of 0.001 M), each for 24 h at 37 °C. Innocuity tests were performed by the World Reference Laboratory (WRL) at The Pirbright Institute.

### Thermo- and pH-stability assay for particles.

The particle stability thermal release assay (PaSTRy) (Walter *et al.*, 2012) was performed in 96-well PCR plates using an Agilent MX3005 PCR machine. Hydrophobicity assays were performed using 1.2 µg of virus and SYPRO orange (Molecular Probes, Invitrogen; final dilution 1 : 2500). RNA release assays were performed using 0.4  µg of virus and SYBR green-II dye (Molecular Probes, Invitrogen; final dilution 1 : 1000). The temperature was ramped from 25 °C to 94 °C in 0.5 °C increments with intervals of 10 s for all assays. Fluorescence was read with excitation and emission wavelengths of 490 nm and 585 nm, respectively, for hydrophobicity assays, and 490 nm and 516 nm, respectively, for RNA release assays. Data sets exported from the qPCR machine were visualized using MxPro software (Stratagene). The exposure of hydrophobic residues (hydrophobicity assay), or the release of RNA and hence the dissociation of capsids (RNA release assay), were detected by increases in fluorescence signal and the melting temperatures (*T*_m_ and *T*_r_, respectively) were taken as the minimum of the negative first-derivative of the fluorescence curve.
